# Primary Cecal Squamous Cell Carcinoma: A Case Report of a Rare Tumor with Poor Prognosis

**DOI:** 10.1155/2021/6680702

**Published:** 2021-07-14

**Authors:** Mohamed Bouzroud, Mustapha Azzakhmam, Aboulfeth El Mehdi, Bouchentouf Sidi Mohammed, El Kaoui Hakim, Oukabli Mohaned, Bounaim Ahmed

**Affiliations:** ^1^Surgery Department (I), Military Hospital, Rabat, Morocco; ^2^Pathology Department, Military Hospital, Rabat, Morocco

## Abstract

Squamous cell carcinoma of the colon is a rare tumor and primary cecal localization is unusual. This malignant condition is marked by a worst prognosis due to early local invasion. We report a case of a 46-year-old female patient admitted to the emergency department with symptoms of peritonitis. CT scan showed a cecal tumor perforated in the retroperitoneal space. The patient underwent right hemicolectomy with D2 lymphadenectomy without intestinal anastomosis. The diagnosis of squamous cell carcinoma was confirmed by histopathological examination. Squamous cell carcinoma is a malignant tumor with poor prognosis, hence, the interest of early diagnosis and management.

## 1. Introduction

Primary squamous cell carcinoma (SCC) of the colon is a very rare malignant tumor with an incidence estimated at 0.1% [1]. Cecal localization is exceptional and characterized by early local invasion and poor prognosis. We present a case of cecal SCC in a 47-year-old female who presented to the emergency department with symptoms of peritonitis. Surgical resection was done, however, the patient died before adjuvant therapy could be offered. This rare entity merits further attention because of his rapid progression and worse prognosis. This case has been reported in line with the SCARE criteria [2].

## 2. Case Report

**Figure 1:**
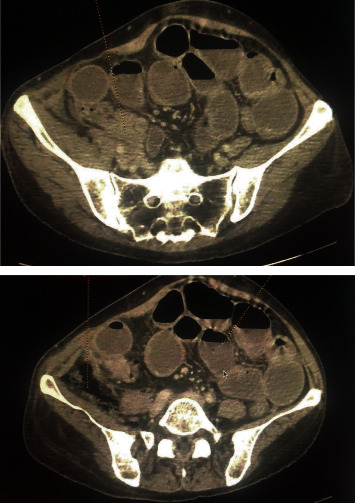
CT scan showing cecal tumor with small bowel distension and air in the retroperitoneal space.

**Figure 2:**
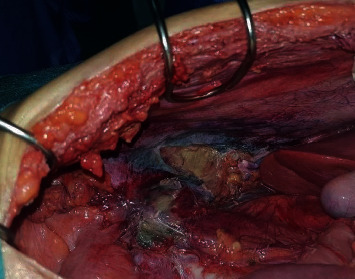
Operating view showing signs of retroperitoneal perforation after right colectomy.

A 46-year-old female patient without medical history presented to the emergency department with symptoms of peritonitis. She was confused and suffered from a huge abdominal pain associated with nausea, vomiting, and fever since 24 hours. Physical examination found diffuse abdominal tenderness with normal vital signs. CT scan revealed signs of peritonitis with psoas abscess and free air in the retroperitoneal space (Figure 1). Emergency laparotomy was performed and surgical exploration revealed a cecal tumor perforated in the retroperitoneum without liver or ovarian metastases. The patient underwent a right hemicolectomy with central ligation of mesenteric vessels and lymph node dissection. Intestinal anastomosis was not performed due to the high risk of leak (Figure 2).

**Figure 3:**
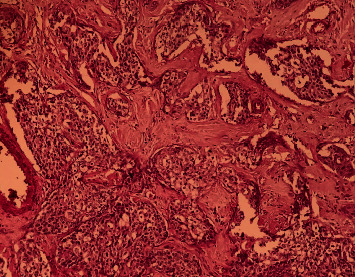
Low magnification HE stain showing tumor proliferation organized in solid islands infiltrating the chorion.

**Figure 4:**
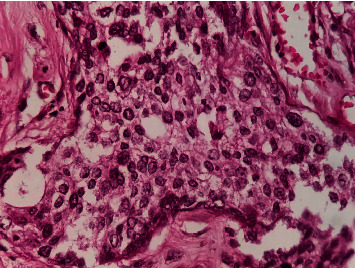
high magnification HEx40, polygonal tumor cells with moderately atypical nuclei and abundant eosinophilic cytoplasm.

**Figure 5:**
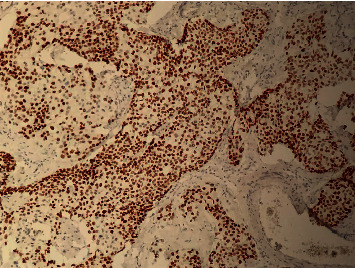
Immunohistochemistry HEX20 intense nuclear marking of tumor cells at P63.

**Figure 6:**
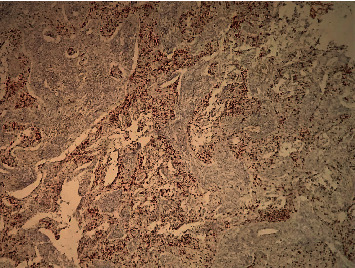
HEx20 Ki67 estimated to more than 9%.

Anatomopathological exam found a white solid cecal tumor measuring 5 × 2 cm with areas of necrosis. Proximal and distal margins were free from tumor, and two of the seventeen nodes dissected were positive. Multiple sections showed a moderately differentiated keratinizing squamous cell carcinoma involving the entire cecal wall. Tumor cells were positive for P63 and negative for neuroendocrine tumors biomarkers (Chromogranin A and Synaptophysin) with a Ki-67 proliferation index estimated to 90% (Figures 3–6). The patient has not received adjuvant chemotherapy because she died from cardiovascular complications of sepsis fifteen days after surgery.

## 3. Discussion

Primary squamous cell carcinomas (SCC) of the colon are extremely rare, accounting for less than 0.5% of all malignant colorectal tumors, with an incidence estimated at 0.1% [1, 3]. Since Schmidtmann reported the first case of colonic SCC in 1919 [4], the number of cases mentioned in the literature has not exceeded 114 according to Sameer et al. [5]. Like our case, this rare tumor mainly affects women in their fifth decade (aged 39 to 60) with a female to male ratio (5 : 1) [6]. The rectum is the usual location of this rare tumor followed by the sigmoid colon [7]. However, the cecum is exceptionally affected, and our case is the third case to report a purely cecal location of a SCC [1].

Till date, the pathogenesis of primary SCC is unknown, and several theories have been suggested to explain the location into the gastrointestinal tract: chronic inflammatory process (ulcerative colitis, infections with Schistosoma or Entamoeba histolytica) may cause a squamous metaplasia, from which carcinomas are developed [6, 8]. Other hypothesis supposes that SCC will originate from a squamous differentiation of pluripotent stem cells [6, 7] or from an existing adenomas or adenocarcinomas [6, 9].

Symptoms of colonic SCC and adenocarcinoma are similar. It depends essentially on the tumor location. However, SCC is more aggressive and can be discovered with some complications: peritonitis due to perforated tumor (like our case), bowel obstruction, or metastases.

Some criteria are necessary to retain the diagnosis of a pure colonic SCC: absence of other locations of squamous cell carcinoma that may cause colonic invasion or metastasis, histological analysis must also rule out an adenosquamous colonic tumor associating glandular and squamous contingents [10].

Symptoms of colonic SCC are identical to those found in other colic tumors: abdominal pain, rectal bleeding, and weight loss. However, due to the aggressive nature of SCC, as in our case, some patients may present symptoms of peritonitis or bowel obstruction.

Surgical resection with regional lymph nodes dissection remains the best way to manage localized colonic SCC. The benefit of systemic chemotherapy or radiotherapy is unclear and still controversial because of the rarity of those tumors and the absence of clinical trial data. Patients, cited in literature, had been treated with an association of 5-fluorouracil and mitomycin or cisplatin [8] or even a combination of adjuvant chemoradiotherapy but without evident benefits.

The prognosis of pure colonic squamous cell carcinoma is worse than adenocarcinoma. The five-year survival rate after surgical resection does not exceed 30% with 80% of recurrences within 3 years [1]. This can be explained by the aggressive nature of these tumors, the late diagnosis, and the absence of well-defined strategies for their management.

## 4. Conclusion

Squamous cell carcinoma of the cecum is a rare entity with a poor prognosis due to the fast local evolution. Early diagnosis and carcinological resection are the hope of patients suffering from it.
